# A Chinese Case of Cornelia de Lange Syndrome Caused by a Pathogenic Variant in *SMC3* and a Literature Review

**DOI:** 10.3389/fendo.2021.604500

**Published:** 2021-09-30

**Authors:** Ran Li, Bowen Tian, Hanting Liang, Meiping Chen, Hongbo Yang, Linjie Wang, Hui Pan, Huijuan Zhu

**Affiliations:** ^1^ Key Laboratory of Endocrinology of National Health Commission, Department of Endocrinology, Peking Union Medical College Hospital, Chinese Academy of Medical Science and Peking Union Medical College, Beijing, China; ^2^ Department of Internal Medicine, Peking Union Medical College Hospital, Chinese Academy of Medical Science and Peking Union Medical College, Beijing, China

**Keywords:** *SMC3*, Cornelia de Lange syndrome, short stature, growth disorders, heterozygous pathogenic variants

## Abstract

**Purpose:**

Cornelia de Lange syndrome (CdLS) is a rare congenital developmental disorder, and cases caused by variants in *SMC3* are infrequent. This article describes a case of CdLS related to a pathogenic variant in *SMC3* and performs a literature review.

**Methods:**

We collected clinical data and biological samples from a 12-year-old boy with “short stature for 11 years”. Gene variants in the proband were detected by whole-exome sequencing, and the variants in his parents were verified by Sanger sequencing. All *SMC3*-related CdLS patients from the PubMed and Web of Science databases were collected and summarized using the available data.

**Results:**

A pathogenic variant in *SMC3* in the proband, c.1942A>G, was identified. Neither of his parents carried the same variant. Twenty-eight patients were diagnosed with CdLS with variants in *SMC3*, including the cases in this study and those reported in the literature, where half of the variant types were missense, followed by 32% (9/28) with a deletion and 11% (3/28) with a duplication. All patients showed symptoms of verbal development delay and intellectual disability to different degrees, and 90% patients had long eyelashes while 89% patients had arched eyebrows.

**Conclusion:**

This study summarized different gene variants in *SMC3* and the frequencies of the various clinical manifestations according to the reported literature. For CdLS caused by *SMC3* variants, short stature and facial dysmorphic features are the two most important clinical clues. Definite diagnosis of this rare disease may be challenging clinically; thus, it is significant to use molecular diagnosis.

## Introduction

Cornelia De Lange syndrome (CdLS) is a rare multisystem congenital developmental disorder inherited in an autosomal dominant manner or X-linked manner (1). Its predominant features include craniofacial malformations, stunted growth, cognitive impairment, behavioral abnormalities, limb deformities and internal organ changes such as gastroesophageal reflux. CdLS was first reported in two infants by Dutch pediatrician Cornelia de Lange in 1933 (2). The reports of its global prevalence are inconsistent. Kline reported it affected approximately 1~3/10000 (3), while Barisic revealed that its prevalence is approximately 1.6~2.2/100000 (4). To some extent, not all individuals with CdLS exhibit the abovementioned typical clinical characteristics, which are complex and diverse, with varying degrees of severity, so the exact incidence and prevalence still need further study (5).

The cohesin complex is a protein consisting of four core subunits, namely, *SMC1A, SMC3, RAD21*, and *STAG*, which form a circular structure that encircles chromatin (6). The complex remains evolutionarily conserved from prokaryotes to eukaryotes. The cohesin complex is mainly involved in the regulation of gene expression, forming DNA loops with CCCTC binding factor (CTCF) and maintaining the normality of chromosomal domains (6). In addition, in the cell cycle, factors such as *NIPBL* and *HDAC8* assist the cohesin complex in maintaining its normal function. In addition, it is responsible for the stability of the genome by participating in the DNA double-strand mismatch repair mechanism, ensuring proper chromosome separation during mitosis and meiosis (7).

Due to the continuous development of gene sequencing technology, the pathogenesis of CdLS has been attributed to variants of genes that encode the structure and regulatory factors of the cohesin complex. Some of the CdLS-related genes have been identified, including *SMC1A*, *SMC3*, *RAD21*, *NIPBL*, *HDAC8*, *BRD4* and *ANKRD11* (8). According to related studies, CdLS has obvious genetic heterogeneity. All of the previously mentioned genetic variants can only explain a small proportion of the clinical cases (3). There are also some typical CdLS patients who do not have the previously mentioned gene variants. In addition, some studies have concluded that the clinical manifestations of CdLS are related to the different kinds of genetic variants that cause the disease. Among them, truncated pathogenic variants in the *NIPBL* gene cause the most serious phenotype, and the phenotype associated with missense variants in *NIPBL* and *SMC3* is relatively milder (6).

This study reported a case of CdLS and summarized all reported cases caused by SMC3 variants to obtain an in-depth comprehension of this rare disease.

## Patient and Methods

### Patient

The patient was 11.7 years old and was admitted to Peking Union Medical College Hospital with the main complaint of “short stature”. Because of his special physical signs, he underwent whole-exome sequencing (WES) after a routine evaluation of thyroid function, growth hormone and bone age. He was found to carry a pathogenic variant in SMC3 and was thus diagnosed with CdLS. The parents signed informed consent forms regarding the research conducted on this boy. This study was performed with the approval of the Ethics Committee of Peking Union Medical College Hospital (JS-1663) and was conducted in accordance with the Declaration of Helsinki.

### DNA Extraction and Whole-Exome Sequencing

We collected approximately 2~3 ml of peripheral blood from the boy (EDTA anticoagulation), and then peripheral blood DNA was extracted by using a Blood DNA Midi Kit (Omega D3494-04, Biotek, USA). To make a precise diagnosis, we performed WES on the proband. Using 3 µg of genomic DNA from each subject for detection, we sheared the DNA into 100~500 base pairs (bp) with a Covaris LE220 ultrasonicator (Massachusetts, USA), and then fragments measuring 150 to 200 bp were picked out by magnetic beads. An adaptor-ligated library that was quality controlled by an Agilent 2100 Bioanalyzer was set up for each individual subject. The cyclized library was continuously sequenced for 50 cycles by a BGISEQ-500 high-throughput sequencer (BGI, Shenzhen, China), and the raw sequencing data were read. Sequencing reads were aligned to a reference human genome (HG19/HG20) using Burrows-Wheeler Aligner (BWA) software, and single nucleotide variants and insertions and deletions were detected by Genome Analysis Toolkit 4.0 (GATK) software, followed by alignment of the database (NCBI dbSNP, HapMap, the 1000 Genomes dataset and a database of 100 healthy Chinese adults) screened for suspicious variants.

### Verification of the *SMC3* Pathogenic Variant by Sanger Sequencing

Primers for our patient’s *SMC*3 variant located in exon 18 were designed by the online tool ‘Primer-BLAST’ (https://www.ncbi.nlm.nih.gov/tools/primer-blast/) based on the *SMC3* reference sequence (NG_012217.1) obtained from NCBI. Forward and reverse primers for *SMC3* (c.1942A>G) were 5’-CAGGGAAAACGCCAATCGTT-3’ and 5’-TGCATACAGCTCAACTGACA-3’.

Total polymerase chain reaction systems containing 10 µl of Taq SuperMix (2X reaction buffer, Taq DNA polymerase dNTPs), 6 µl of double-distilled water, 2 µl of genomic DNA, and 1 µl of forward and reverse primers (10 µmol/L) were amplified. The cycling conditions were 94°C for 4 minutes; 35 cycles of 94°C for 30 seconds, appropriate annealing temperatures for 30 seconds and 72°C for 30 seconds; and one cycle at 72°C for 4 minutes followed by storage at 4°C. The pathogenicity of the *SMC3* variant in this boy was graded according to the American College of Medical Genetics and Genomics (ACMG) guidelines (9).

### Literature Review and Statistical Analysis

All cases of CdLS (up to February 1st, 2020) were collected from the PubMed and Web of Science databases using the keywords “Cornelia de Lange syndrome”, and the identified papers were carefully reviewed to include all relevant papers (9–16). The clinical manifestations and genetic results of all cases reported were analyzed and summarized by using SPSS version 25.0.

## Results

### Case Report

An 11.7-year-old boy was admitted to our hospital with the chief complaint of short stature. His birth weight was 2.45 kg (-2.15 standard deviation score, SDS), and his birth length was 49 cm (-0.79 SDS). He underwent full-term natural delivery and lived in a neonatal incubator for a week due to pyloric spasm. The maternal pregnancy history was unremarkable. In his daily life, he had hyperactivity disorder and poor academic performance, and his pronunciation was vague. He often had enuresis at night.

One year after birth, his height was found to be shorter than normal without detailed records. At the age of 5.5 years, he underwent a growth hormone stimulating test, and the results showed that his growth hormone (GH) baseline was 3.54 ng/ml, and the GH peak value was 8.49 ng/ml. His results of insulin-like growth factor-1 (IGF-1) and insulin-like growth factor binding protein-3 (IGFBP-3) were all within the normal ranges. Therefore, he was diagnosed with partial growth deficiency and was treated with recombinant human growth hormone (rhGH) (unknown dosage). The treatment lasted for 3 months, which resulted in a height increase of 3 cm but it was stopped due to the high cost of the therapy. His pronunciation was nebulous.

**Figure 1:**
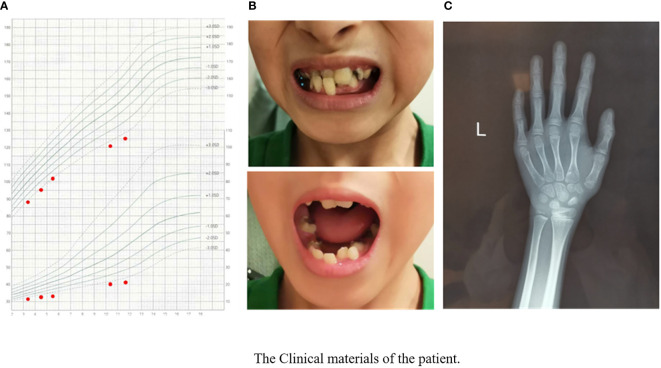
The clinical materials of the patient’s growth chart. **(A)** The manifestation of the patient’s teeth. **(B)** X-ray of the patient’s left hand and wrist. **(C)** Bone age was approximately 7 years when the patients’ chronological age was 11 years 8 months.

The physical examination results when visiting our outpatient clinic were as follows: his height was 125.4 cm (-3.3 SDS), and his weight was 21 kg (-3.2 SDS). The growth curve of the patient is shown in **Figure 1A**. His dentition was very disorderly (**Figure 1B**). He had a thin body, slender limbs, a head circumference of 48 cm, an armspan of 132 cm, and clinodactyly of the 5th finger. HbA1c, thyroid function tests, liver and renal function tests and routine blood tests were normal. His IGF1 level was 133 ng/ml (reference range, RR: 111~551 ng/m, -1.8 SDS). His bone age was approximately 7 years old while his chronological age was 11.7 years (**Figure 1C**).

The parents denied consanguinity. His father was 180 cm tall, and his mother was 155 cm tall. The height of his 18-year-old brother was 176 cm.

### Genetic Study

**Figure 2:**
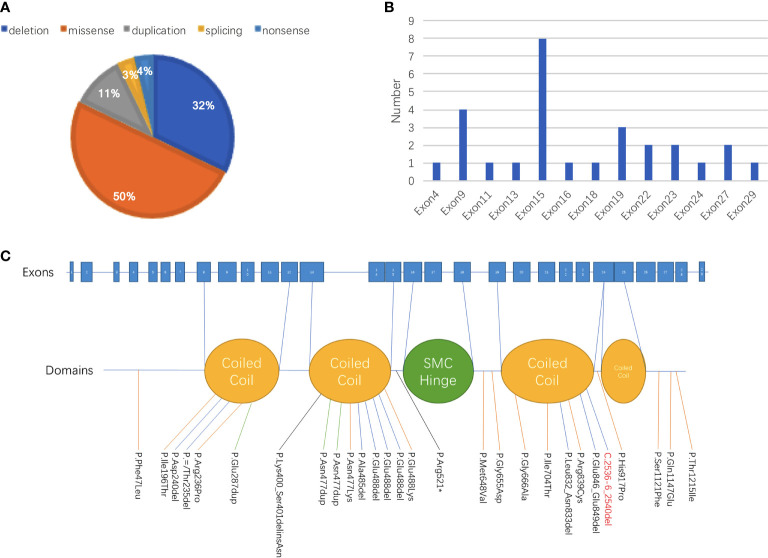
Summary of genetic abnormalities in patients with CdLS carrying *SMC3* pathogenic variants. **(A)** Among all 28 patients with records of genetic alterations, half of the variant types were missense, followed by deletion (32% [9/28]) and duplication (11% [3/28]). One patient was discovered carrying a splicing variant, and one patient was detected with a nonsense variant. **(B)** The site with the largest proportion of variants in *SMC3* was exon 15, which was 28.6%, followed by variants that occurred in exon 9, exon 19, exon 22, exon 23, and exon 27. **(C)** The proportion of patients whose variants were in the domain named coiled coil was 60% (12/20), and 40% (8/20) of patients had alterations in other parts.

His karyotype was normal (46,XY). The WES results of the proband were as follows: a heterozygous variant in exon 18 of *SMC3*, c.1942A>G (p. Met648Val), was detected. This pathogenic variant of *SMC3* is a missense variant that causes CdLS-3 (MIM 610759). After receiving the genetic results of the proband, the corresponding variant sites of the parents were checked. The results of *SMC3* gene Sanger sequencing showed that neither of his parents carried the same variant in *SMC3* (**Figure 2**).

### Literature Review

Due to the rapid development of genetic testing technology, CdLS has been reported in many cases with variants in various genes and different sites since the first case of CdLS was reported in 1933 (2). To date, it is widely believed that CdLS is primarily caused by variants in seven genes, including *NIPBL*, *SMC1A*, *SMC3*, *RAD21*, *BRD4*, *HDAC8* and *ANKRD11*. Through February 1, 2020, 27 cases of CdLS with definite variants of *SMC3* were reported (**Tables 1** and **2**), including 22 cases with detailed clinical data including sex and age. None of these cases were reported in a consanguineous family. The male to female ratio was 1.44 (13:9) from the available data. In regard to the age distribution of the patients, 6 of 21 (28.6%) people with a recorded age were adults, and 8 of 21 (38.1%) were infants whose ages were between one and three. The remaining 7 patients ranged from 3 to 18 years old. The patients’ mean age when they were diagnosed was 10.8 ± 9.7 years old. For the recorded gestational weeks of 18 patients, including the case reported in our center, 14 patients were full-term deliveries, and 4 patients were premature, of which two had the shortest gestational weeks, only 35 weeks. One article reported that among patients with prenatal CdLS, symmetric intrauterine growth restriction (IUGR) with onset in the second trimester was the most common finding significant for the diagnosis of CdLS (17). However, there were no records about the birth weight or length of these patients, which suggests that we need to pay extra attention to such infants.

**Table 1:** Frequency of Clinical Features in Individuals with *SMC3* variants.

Clinical Characteristics	Frequency
Brachycephaly	58% (11/19)
Low anterior hairline	50% (9/18)
Microcephaly	47% (9/19)
Arched eyebrows	89% (17/19)
Synophrys	74% (14/19)
Thick eyebrows	71% (12/17)
Long eyelashes	90% (18/20)
Depressed nasal bridge	44% (8/18)
Anteverted nostrils	53% (9/17)
Long and/or featureless philtrum	56% (10/18)
Broad/bulbous nasal tip	76% (13/17)
Thin upper lip vermilion	70% (14/20)
Downturned corners of mouth	53% (10/19)
Micrognathia/retrognathia	53% (10/19)
Small hands	67% (12/18)
Proximally set thumbs	70% (14/20)
Short first metacarpal	61% (11/18)
Clinodactyly fifth finger	61% (11/18)
Short fifth finger	71% (12/17)
Hirsutism	84% (16/19)
Small feet	69% (11/16)
Syndactyly of toes	78% (14/18)
Restriction of elbow movements	50% (6/12)
Cardiac defects	61% (11/18)
Feeding problems in infancy	80% (12/15)
Hearing loss	53% (8/15)
Verbal developmental delay	100% (19/19)
Intellectual disability	100% (13/13)

**Table 2:** Summaries of clinical phenotype and *SMC3* variants of the 28 patients with Cornelia de Lange syndrome.

	Num	Age	Gender	Severity	Birth weight (kg)	Birth length (cm)	Weight kg (SDS)	Height cm (SDS)	Head	Nose	Eyes	Mouth	Hand	Feet	Developmental delay	Variant site	Type of variant	Exons
Yuan et al. (15)	P1	17y	M	moderate	/	/	/	/	+(microcephaly)	/	+(TE、Sy、long curly eyelashes、 strabismus)	+(TUL、M/R)	+(moderate brachydactyly, C5F)	/	+	c.2536-2547del	deletion	23
Kaur et al. (12)	P2	/	M	/	/	/	/	/	/	/	/	/	/	/	/	c.1464_1466del	deletion	15
Yuan et al. (15)	P3	/	/	/	/	/	/	/	/	/	/	/	/	/	/	c.587T>C	missense	9
	P4	/	/	/	/	/	/	/	/	/	/	/	/	/	/	c.1453-1455del	deletion	15
	P5	/	/	/	/	/	/	/	/	/	/	/	/	/	/	c.2111T>C	missense	19
	P6	/	/	/	/	/	/	/	/	/	/	/	/	/	/	c.3362C>T	missense	27
	P7	/	/	/	/	/	/	/	/	/	/	/	/	/	/	c.2536-6_2540del:splicing	splicing	23
	P8	/	/	/	/	/	/	/	/	/	/	/	/	/	/	c.717_719del	deletion	9
Dowsett et al. (11)	P9	2y	M	/	/	/	/	/	/	/	/	/	/	/	+	c.1431T>A/G	missense	15
Infante et al. (10)	P10	7m	F	/	2.24	47	5.89(1st%)^*^	62(<1st%)^*^	+(microcephaly、bitemporal narrowing of forehead, LAH)	+(DNB, AN)	+(AE, Sy, TE, LE, HOL, P, bilateral epicanthal folds)	+(DCOM, micrognathia, SN)	+(PST, C5F, S5F)	+(SOT)	+	c.1433-1435dup	duplication	15
	P11	22y	F	/	3.17	/	95.25	170.6	+(narrow forehead, LAH)	+(BNT)	+(AE, Sy, TE, LE, P, M, bilateral epicanthal folds)	+(DA-small widely spaced teeth, micrognathia)	+(SH, PST, S5F)	+(SOT)	+	c.1433-1435dup	duplication	15
Gil-Rodriguez et al. (13)	P12	26y	F	mild	2.8(-1.40)	/	58(-1.80)	150(-2.20)	+(B, LAH)	+(L/FP, BNT)	‘+(AE, Sy,LE)	+(TUL, DCOM, DA, SN)	+(SH, C5F, S5F)	+(SF, SOT)	+	c.139T>C	missense	4
	P13	2.5y	F	moderate	2.865(-1.23)	/	11(-1.46)	86(-1.10)	+(B, microcephaly)	+(DNB, AN-mild, BNT-mild)	+(AE, Sy, LE, M, HOL-mild)	+(TUL, DCOM, PHA, M/R, SN)	+(PST, SFM-mild, C5F-mild, S5F)	/	+	c.[=/703_705del] mosaic	deletion	9
	P14	4.5y	M	mild	2.67(-1.98)	49.5(-0.8)	14.5(-1.6)	104(-0.33)	‘+(B, M, posterior hair whorl on left side)	/	+(AE,LE)	+(TUL, caries)	+(PST, SPC- right interruped palmar crease)	/	+	c.707G>C	missense	9
	P15	2m	M	moderate	2.27(-2.91)	44.5(-3.84)	3.6(-3.02)	50.3(-3.6)	+(LAH)	+(AN, L/FP, BNT)	+(AE, Sy, TE, LE),	+(TUL, DCOM,M/R)	+(SH, PST, SFM)	/	+	c.859_861dup	dup	11
	P16	23y	F	severe	2.14(-2.2)	44(-2.81)	42(-5.3)	137(-4.40)	+(LAH)	+(AN, L/FP, BNT)	+(AE, TE, LE, P, M, lateral extension eyebrows)	+(TUL, DCOM, SN-Klippel-Feil, lowset anteverted ears)	+(SH, PST, SFM, C5F, S5F, SPC)	+(SF, SOT-2~3)	+	c.1200_1202delGTC	deletion	13
	P17	29y	M	mild	2.19(-2.31)	48(-0.8)	37(-6.8)	147(-4.5)	/	+(BNT)	+(AE, Sy, TE, LE, M)	+(TUL, PHA),	+(SH, PST, SFM, C5F, S5F, SPC)	+(SF, SOT-2~3)	+	c.1464_1466delAGA	deletion	15
	P18	21y	M	moderate	2.57(-2.23)	/	39.5(-5.6)	150.8(-3.8)	+(Sh-thick)	+(DNB, AN, BNT)	+(AE, Sy, TE, LE, deepest eyes)	+(DCOM?, Prognathism)	/	‘+(SF)	+	c.1464_1466delAGA	deletion	15
	P19	11y6m	M	mild-moderate	2.39(-0.1)	44(-1.1)	26(-1.74)	136(-1.74)	+(B, LAH, M, Low, frontal hairline, Sh-thick)	+(DNB, AN, L/FP, BNT)	+(AE,TE,LE)	+(TUL, PHA, DA, SN, Facial asymmetry, low-set ears)	+(SH, PST, SFM, C5F)	+(SF)	+	c.1462G>A	missense	15
	P20	8y7m	F	mild	2.94(0.72)	/	24(-0.85)	120(-1.80)	/	+(DNB, L/FP)	+(Sy, LE)	+(M/R)	/	/	+	c.1561C>T	nonsense	16
	P21	33m	F	moderate	2.1(-1.79)	42(-2.2)	10.22(-2.56)	79(-3.7)	+(B, microbrachycephaly)	+(AN, L/FP, BNT)	+(AE, LE, LDO, astigmatism)	+(TUL, PC)	+(SH, SFM, C5F)	+(SF)	+	c.1964G>A	missense	19
	P22	22y	M	mild-moderate	3.21(-2.48)	49(-0.83)	63(-1.02)	163(-2.26)	+(B)	+(L/FP, BNT)	+(AE, LE, Sy, LE, M, Exotropia)	+(TUL, DCOM, PC-soft, palate, DA-widely spaced)	+(SH, PST, SFM, C5F)	+(SF)	+	c.1997G>C	missense	19
	P23	11m	M	moderate	2.985(-1.22)	49(-0.83)	7.5(-2.27)	71(-0.68)	+(B, LAH)	+(AND, AN, L/FP)	+(AE, LE, P, M, LDO)	+(TUL, DCOM, PHA, DA-delay, M/R, SN-low, posterior hairline)	+(SH, PST, SFM, C5F, S5F-bilateral dysplasia of the middle phalanx of the fifth finger)	+(SF)	+	c.2494_2499del	deletion	22
	P24	6y	M	severe	3.005(-1.17)	/	18(-1.19)	104(-2.46)	+(B, plagiocephaly, frontal bossing)	+(BNT)	+(AE, TE, LDO),)	+(DA-dysmorphic teeth, pegged incisors, caried, mild low-set and posteriorly rotated ears, small mouth, delayed closure of anterior fontanelle (21m), prognathism, flat facies)	+(SH, PST, SFM, S5F, SPC)	+(SF)	+	c.2515C>T	missense	22
	P25	29m	F	moderate	2.18(-1.6)	44.45	10(-2.64)	82(-2.09)	+(B, LAH, Microcephaly, SH)	+(L/FP-smooth, not long)	+(Sy, TE, LE, P, LDO, exotropia, astigmatism)	+(TUL, DCOM)	+(SH, PST)	+(SF)	+	c.2750A>C	missense	24
	P26	12y5m	M	moderate	2.778(-1.7)	48(-1.5)	20(6.9y)(-0.91)	110(6.9y)(-2.05)	+(b, SH-thick)	+(DNB, AN, BNT)	+(AE, Sy, LE, P, HOF)	+(PHA, M/R, SN, Mild low-set and posteriorly rotated ears)	+(SH, PST, SFM, C5F, S5F)	+(SF, SOT-2~3)	+	c.3439C>G	missense	27
	P27	5m	F	severe	/	/	2.57(-7.51)	48(-6.8)	+(B, LAH, microcephaly, Sh-thin)	+(DNB, L/FP, BNT)	+(AE,Sy, TE, LE)	+(TUL, DCOM, M/R, growly cry)	+(SH, SFM, S5F)	/	+	c.3644C>T	missense	29
The current case	P28	12y	M	mild	2.45	49	21	125.4	+(microcephaly, skull)	/	+(AE, LDO, bilatheral epicanthal foldings)	+(DA-Widely spaced teeth, uneven teeth),	+(C5F, S5F)	/	+	c.1942A>G	missense	18

*This case did not indicate the specific age at this time, only marked as 1st% in the report.

B, brachycephaly; LAH, low anterior hairline; S, skull; Sh, scalp hair; AE, arched eyebrows; Sy, synnophrys; TE, thick eyebrows; LE, long eyelashes; P, ptosis; M, myopia; LDO, lacrimal duct obstruction; HOL, hooding of lids; DNB, depressed nasal bridge; AN, anteverted nostrils; L/FP, long/featureless philtrum; BNT, broad/bulbous nasal tip; TUL, thin upper lip; DCOM, downturned corners of mouth; PHA, palate high arch; PC, palate cleft; DA, dental anomalies (small/widely spaced); M/R, micrognathia/retrognathia; SN, short neck; SH, small hands; PST, proximally set thumbs; SFM, short first metacarpal; C5F, clinodactyly 5th finger; S5F, short 5th finger; SPC, Single Palmar crease; SF, small feet; SOT, syndactyly of toes.

Detailed clinical characteristics of all of the patients are listed in **Table 2**. Verbal developmental delay and intellectual disability were found in all patients. Other clinical manifestations with relatively higher frequencies include long eyelashes (90% [18/20]), arched eyebrows (89% [17/19]), hirsutism (84% [16/19]), feeding problems in infancy (80% [12/15]), syndactyly of toes (78% [14/18]), broad/bulbous nasal tip (76% [13/17]), synophrys (74% [14/19]), short fifth finger (71% [12/17]), thick eyebrows (71% [12/17]), proximally set thumbs (70% [14/20]) and thin upper lip vermilion (70% [14/20]). In the current case, our patient showed a suspected condition of chronic constipation, which has been reported in the previous literature (5). However, there was no available information about constipation in other cases of patients with *SMC3* variants. According to the evaluation standard stated in a previous study, 20 patients with detailed records of their clinical manifestations were rated. According to the classification of the disease’s severity raised by Kline et al., only 3 of them reached severe levels, 10 of them reached moderate levels, and 7 of them were regarded as mild levels (18). However, among the clinical data we obtained thus far, biochemical data and imaging auxiliary examination data are extremely limited.

Except for a baby girl whose variant was inherited from her mother, there were 18 patients with *de novo* variants as confirmed by parental verification. The proportions of different types of variants are shown in **Figure 2**. Among all 28 patients with records of genetic alterations, half of the variant types were missense (50% [14/28]), followed by deletion (32% [9/28]) and duplication (11% [3/28]). Additionally, there was one patient with splicing variant and one patient with nonsense variant. At the same time, we have an idea of the number of variants in each exon as shown in **Figure 2B**. The number of variants that occurred in exon 15 accounted for the largest proportion (28.6%), followed by variants that occurred in exon 9, exon 19, exon 22, exon 23, and exon 27. Different variants corresponding to different protein domains are listed in **Figure 2C**. The proportion of patients whose variants were located in the coiled-coil domain was 60% (12/20), and patients whose alterations were located in other parts accounted for 40% (8/20).

## Discussion

When a short stature patient has multisystem involvement, such as limb deformities, organ dysplasia, and cognitive and behavioral defects, it is necessary to pay attention to possible genetic diseases. With the development of gene sequencing technology, Sanger sequencing, panel detection and WES will help us identify the underlying genetic abnormalities and make a molecular diagnosis of a specific genetic disease. As an important type of dwarfism caused by a single gene variant, CdLS is relatively rare, but if the patient has typical facial features, poor language and mental development, or various hand deformities, we ought to consider its possibility.

In this case, the patient’s long arms attracted our attention. Although his height was 125.4 cm, he had a 132 cm armspan at that time. To our knowledge, symptoms of a significantly longer armspan than height have never been reported in patients carrying variants in *SMC3*; therefore, careful physical examination provides valuable clues in diagnosing genetic disease. Additionally, this patient had a unique manifestation of his dental abnormalities; although there was wide space between his teeth, some teeth overlapped and squeezed together, which might be related to his micrognathia. Based on the data we collected, verbal development delay and intellectual disability were the two most common symptoms of this condition, which were found in all of the patients to varying degrees. There was one patient who had not spoken for decades, and some patients only showed a certain degree of slurring. Therefore, an evaluation of intellectual development and language development should be given special attention in clinical settings. If necessary, development-related tests such as systematic intelligence tests can be performed instead of simple evaluations. In addition, changes in the facial characteristics of patients with CdLS, such as arched eyebrows, long eyelashes, and synophrys, have a high incidence, occurring in 90% of the patients, and micrognathia is another common feature. Since approximately 50% of patients with micrognathia can be detected by prenatal ultrasound examinations (19), this feature should be given extra attention during prenatal examinations by clinicians. Additionally, hirsutism, syndactyly of the toes, and feeding problems in infancy are also symptoms that can be seen frequently and need to be called out for special attention.

On the basis of reviewed data, it was obvious that *SMC3* variants occurred mostly in exon 15, accounting for 28.5% (8/28) in all of the *SMC3* variants reported, followed by variants that occurred in exon 9, exon 19, exon 22, exon 23, and exon 27 (**Figure 2**). The c.1942A>G (p.Met648Val) variant in the current case was the first variant occurred in exon 18, which was never been reported before. Three unrelated cases were found to have variants in the same position (c.1464_1466del). Two of these three patients had detailed information: one was evaluated as moderate, and the other was graded as mild. Patients carrying the same *SMC3* variant presented with various degrees of severity, demonstrating the phenotypic heterogeneity caused by *SMC3* variants. Heterogeneity appears not only in *SMC3* but also in *NIPBL*. One study pointed out that unrelated probands with a similar *NIPBL* variant can have a phenotype ranging from severe to mild (20).

The cohesin complex plays a crucial role in regulating gene expression (21, 22). Cohesin combines with the sequence-specific DNA binding protein CTCF and forms structural topologically associated domains (TADs), chromatin loops and contact domains, which can bring together distant enhancers with promoter sequences to regulate gene expression. Disruption of the cohesin complex leads to abnormal DNA domain topology, resulting in gene expression dysregulation. CdLS is predominantly caused by pathogenic variants of genes that encode the structure and regulatory factors of the cohesin complex. The data show that *NIPBL* variants can be detected in nearly 70% of CdLS patients. Meanwhile, the proportion of patients carrying *HDAC8* variants was approximately 5%, and 5% of patients carried variants in *SMC1A*. CdLS caused by *SMC3* and *RAD21* each accounted for less than 1% of cases (7).

There is a correlation between genotype and clinical phenotype to some extent in patients diagnosed with CdLS. As previously reported, *NIPBL* truncating, nonsense, splice site and frameshift pathogenic variants will produce more truncated and nonfunctional NIPBL proteins, and the NIPBL expression level is the lowest in patients with *NIPBL* variants compared with patients with *SMC3* variants, so the phenotype of patients carrying *NIPBL* variants will be more serious than those carrying *SMC3* variants. Using the severity of the cognitive developmental defects in patients as an example, patients with *NIPBL* variants are mostly moderate to profound and patients with *SMC3* variants are mostly mild to moderate. Similarly, patients with *NIPBL* variants have significantly more severe upper limb deformities than patients with *SMC3*. The former may have forearm loss, while brachydactyly and clinodactyly are more common in the latter. In terms of organ development, the incidence of cardiac, gastrointestinal, and urinary system defects in patients with *SMC3* variants is lower than that in patients with *NIPBL* variants. Interestingly, variants in *SMC3* were even found in some patients who were short and did not meet the diagnostic criteria for atypical CdLS, which includes intellectual and cognitive dysfunction (23).

In our study, only one of the 28 patients (P13) was found to be mosaic, but her phenotype can be classified as moderate according to the rating standard stated in 2007 (18). There was also a report analyzing the relationship between whether *NIPBL* mutant patients are mosaic and the severity of symptoms. The results showed that somatic mosaicism does not seem to be consistently connected with a milder phenotype. According to these reports, nearly 15 to 20% of patients with typical clinical characteristics of CdLS and *NIPBL* variants have the variants in other tissues and not in their lymphocytes (24, 25). Therefore, the international consensus statement suggests that clinicians should study patients’ fibroblast cells, buccal cells, and bladder epithelial cells to confirm whether they are mosaic when variants of related genes cannot be detected in lymphocytes. Although patients with *SMC3* variants have relatively milder clinical symptoms than those with *NIPBL* variants, mosaicism should be taken into consideration in clinical settings.

This patient received rhGH treatment within a very short period and displayed a good effect with an estimated annual growth rate of 12 cm. However, they regrettably ceased the therapy for financial reasons. To date, on the basis of the literature we collected, only one report showed that rhGH had achieved good results in treating patients with short stature caused by CdLS (26). Although these two patients had a good response to rhGH, the number of cases was too small, and whether rhGH can be considered a commonly accepted treatment for patients with CdLS-induced short stature requires further clinical research.

The diagnosis and management of CdLS is challenging because of its multisystemic malformations. For patients with multiorgan involvement, facial dysmorphic features, growth retardation, cognitive disorder, and verbal and behavioral developmental defects, a suspicion of CdLS should be made. Molecular genetic testing assists in making a confirmative diagnosis of CdLS. Once the diagnosis is acquired, lifelong medical care and multidisciplinary and social care are urgently needed to improve the patient’s health and increase their quality of life (3, 27, 28). Interdisciplinary management integrates doctors from different disciplines to establish a treatment team, which enables patients with CdLS to be evaluated from different professional perspectives and it proposes syndrome-specific, individualized treatment plans. The team includes gastroenterologists for gastroesophageal reflux, cardiologists for congenital heart diseases, dentists for dysplasia of the teeth, psychotherapists for cognitive and behavioral defects, orthopedists for the correction of limb deformities, speech specialists for verbal defects, urologists for urinary system abnormalities, and geneticists who provide the necessary genetic counseling to the patients’ parents (28). However, patients seldom have the chance to receive the therapies mentioned above. In the current case, our patient experienced delayed diagnosis and treatment, likely due to a lack of knowledge regarding CdLS by primary hospital caregivers, their geographic isolation and financial considerations. There is a long way to go in educating both caregivers and patients to achieve an early diagnosis, apply early interventions, diminish complications and improve the patients’ quality of life.

## Conclusion

*SMC3* gene variants are a rare cause of CdLS. We reported a 11.7-year-old male carried a *de novo SMC3* variant, c.1942A>G (p.Met648Val), which was the first variant occurred in exon 18. Patients usually have mild to moderate typical clinical manifestations. Some facial features, such as arched eyebrows, long eyelashes and broad/bulbous nasal tips, as well as some intellectual developmental abnormalities and verbal development delay, are common clinical manifestations that require more attention in clinical settings. To our knowledge, the current case is the second case in which rhGH was used to improve the short stature of patients with CdLS. Although the results suggest that rhGH can be used as an effective treatment for the short stature caused by CdLS, more clinical evidence is necessary.

## Data Availability Statement

The data presented in the study are deposited in the SRA repository, accession number PRJNA751153. 

## Ethics Statement

Written informed consent was obtained from the patient’s parents, for the publication of any potentially identifiable images or data included in this article.

## Author Contributions

All authors helped to perform the research. HZ conceived and designed the research. HZ, HP, LW and HY contributed to the project management. HL and MC helped to collect clinical samples. RL did molecular experiments. RL and BT took part in the statistical analysis. RL and BT wrote the manuscript. HY and LW took part in the revision of the manuscript. All authors contributed to the article and approved the submitted version.

## Funding

This work was funded by the CAMS Innovation Fund for Medical Science (CAMS-2016-I2M-1-002, CAMS-2016-I2M-1-008) and the National Key Research and Development Program of China (No. 2016YFC0901501).

## Conflict of Interest

The authors declare that the research was conducted in the absence of any commercial or financial relationships that could be construed as a potential conflict of interest.

## Publisher’s Note

All claims expressed in this article are solely those of the authors and do not necessarily represent those of their affiliated organizations, or those of the publisher, the editors and the reviewers. Any product that may be evaluated in this article, or claim that may be made by its manufacturer, is not guaranteed or endorsed by the publisher.
